# Seasonal Dynamic Changes in the Nutrient Elements and Antioxidant Activity of *Ilex vomitoria* Leaf

**DOI:** 10.3390/plants14131919

**Published:** 2025-06-23

**Authors:** Luqiong Sha, Yanyan Yin, Yilin Xue, Xue Zou, Bingsong Zheng, Jianhong Zhang, Daoliang Yan

**Affiliations:** 1 National Key Laboratory for Development and Utilization of Forest Food Resources, Zhejiang A&F University, Hangzhou 311300, China; lqsha@stu.zafu.edu.cn (L.S.); yinyan@stu.zafu.edu.cn (Y.Y.); xyl@stu.zafu.edu.cn (Y.X.); 19157738591@163.com (X.Z.); bszheng@zafu.edu.cn (B.Z.); 2 Ningbo Academy of Agricultural Sciences, Ningbo 315000, China; nbjianhong@163.com

**Keywords:** *Ilex vomitoria*, nutritional components, aroma components, antioxidant, development and utilization

## Abstract

*Ilex vomitoria* Ait. is a star substitute for “tea” in recent years. At present, research on *I. vomitoria* mainly focuses on its breeding and cultivation, and there are few reports on the seasonal changes of important components such as leaf nutrients. This study focuses on the leaves of the topmost annual branches of *I. vomitoria*. Leaves were harvested at different stages, and the nutrient elements, antioxidant substances, antioxidant capacity, and aroma components in the leaves were measured and analyzed. The results showed that the content of mineral elements, soluble sugars, vitamin C, amino acids, flavonoids, polyphenols, saponins, caffeine, and catechins, as well as the DPPH free radical scavenging ability, ABTS cation free radical scavenging ability, and FRAP iron ion reduction ability in the leaves of *I. vomitoria* showed significant differences with seasonal changes. The mineral element content in spring leaves is relatively high. Flavonoids and polyphenols are the main antioxidant substances in the leaves of *I. vomitoria*, indicating that the antioxidant capacity of spring leaves is the strongest. The content of aroma components in the leaves of *I. vomitoria* in spring is the highest, with alcohols ranging from 54.93% to 66.08%, followed by ketones from 17.63% to 48.07%, and aldehydes from 21.27% to 38.51%. Overall, spring leaves are more suitable for harvesting, development, and utilization.

## 1. Introduction

China’s abundant leaf resources provide an important material foundation for the cycle of material of ecosystems and human survival. However, the overall utilization rate of leaves by humans is relatively low, with most leaves left to fall or abandoned in forest areas after harvesting, and a small portion used as fuel, fertilizer, or feed. Therefore, the rational development and utilization of abundant leaf resources is to meet the needs of human continuous development, especially the development of a green economy.

The leaves of many trees are rich in nutritional value and have a wide range of uses [1]. For example, the leaves of *Diospyros kaki* L. are rich in flavonoids, vitamin C, amino acids, etc. They are often used to make tea and have the effects of clearing heat, lowering blood pressure, and lowering blood lipids. They can also be used as medicine or topically to stop bleeding [2,3]. *Crataegus pinnatifida* Bge. leaves contain a high amount of polyphenols and flavonoids, which play an important role in lowering blood sugar and preventing cardiovascular diseases [4]. *Sapium sebiferum* L. leaves have the effects of reducing swelling, dispersing blood stasis, clearing heat, and detoxifying [5]. Leaves are also an important source of feed. Research has shown that the leaves of *Pteroceltis tatarinowii* Maxim. contain high levels of protein, fat, crude fiber, and other components, which can be used as livestock feed [6]. Meanwhile, Souilem et al. [7] have extracted and separated the material components of olive leaves, and the obtained extracts were used in nutritional and beauty products. Liao et al. have converted pineapple leaves into renewable fuel through processes such as ethanol fermentation for utilization [8].

*Ilex vomitoria* Ait. is an evergreen shrub belonging to the Aquifoliaceae family, native to the southeastern United States. Its leaves and tender branches are traditionally used to make healthy drinks [9,10]. *Camellia sinensis* L. leaves contain abundant caffeine and are a classic beverage in China. However, in recent years, people have been actively seeking new caffeine-rich plant resources to replace conventional beverage plants such as tea. Due to its unique active substance caffeine, *I. vomitoria* has become a star substitute for “tea” in recent years, with huge economic and social benefits, and broad prospects for development and utilization.

At present, research on *I. vomitoria* mainly focuses on breeding and cultivation [11,12], and there are few reports on the seasonal changes of important components such as leaf nutrients during its annual growth cycle. This study thus represents the first comprehensive exploration of the nutritional components, antioxidant components, and aroma components of leaves of different seasons of *I. vomitoria*, and further evaluates the influence of seasons on them. The research results indicate that the nutritional components, antioxidant compounds, and aromatic compounds in *I. vomitoria* leaves vary significantly with the seasons.

## 2. Results

### 2.1. Analysis of Mineral Nutrients in the Leaves of I. vomitoria

The analysis of elemental content in *I. vomitoria* leaves is summarized in Table 1. The difference between the highest and lowest contents of macroelements among the three seasons was 2.87 mg/g DW for potassium (K). Among the macroelements, the variation ranges were 5.14 g/kg for N, 1.17 g/kg for P, 1.58 g/kg for K, 1.19 g/kg for Mg, and 1.73 g/kg for Ca, respectively. The difference between the highest and lowest contents of microelements among the three seasons were 14.27 mg/kg for Fe and 94.17 mg/kg for Zn, respectively.

The contents of N, P, and Fe in spring leaves of *I. vomitoria* leaves were significantly higher than those in summer and autumn (*p* < 0.05), with N content reaching as high as 17.00 g/kg; the Ca content in autumn was significantly higher than that in spring and summer; there was no significant difference in the content of K, Mg, and Zn between spring and autumn (*p* > 0.05); there was no significant difference in the content of K in leaves between spring, summer, and autumn (*p* > 0.05). The above results indicate that tender leaves harvested in spring have abundant mineral elements.

### 2.2. Analysis of Soluble Sugar and Vitamin C Content in Leaves of I. vomitoria

As shown in Table 1, the leaf soluble sugar content was highest in spring and significantly greater than in summer and autumn (*p* < 0.05), with the lowest levels observed in summer. The soluble sugar content in spring and autumn was 65% and 20% higher than in summer, respectively.

The leaf vitamin C content was highest in autumn and significantly greater than in spring and summer (*p* < 0.05), with the lowest levels observed in spring. The vitamin C content in autumn was 70% and 30% higher than in spring and summer, respectively.

### 2.3. Analysis of Amino Acid Content and Its Flavor in Leaves of I. vomitoria

As shown in Table 2, in spring, the highest amino acid was Arg, which was 402.81 µg/g, and the lowest was Cys, which was 20.36 µg/g. In summer, Asp had the highest content at 324.17 µg/g, while Cys had the lowest content at 11.96 µg/g. In autumn, Ile had the highest content at 116.34 µg/g, while His had the lowest content at 9.28 µg/g. The highest total amino acid content in leaves was in spring, at 1645.14 µg/g, followed by summer at 1425.10 µg/g, and the lowest was in autumn, at 554.81 µg/g. The highest content of essential amino acids (EAAs) in *I. vomitoria* leaves was observed in spring (571.62 µg/g dry weight), followed by summer (394.81 µg/g) and autumn (225.19 µg/g) In summary, the content of 17 amino acids in the leaves of *I*. *vomitoria* was highest during spring harvesting and lowest during autumn harvesting, with significant differences between the two (*p* < 0.05).

According to Table 3, in spring, the total TAVs of various flavor amino acids were in the order of umami amino acids > bitter amino acids > sweet amino acids > aromatic amino acids. The total content of amino acids in the spring flavor of *I. vomitoria* leaves was 1601.13 µg/g, with Arg having the highest content at 402.81 µg/g and the highest TAV at 4.03. During spring, the primary contributor to the sweet taste among amino acids was His; to the bitter taste, Arg; to the fresh taste, Glu and Asp; and to the aromatic taste, Cys. Collectively, these five free amino acids (His, Arg, Glu, Asp, and Cys) were the key determinants of the unique flavor profile in spring *I. vomitoria* leaves.

In summer, the order of total TAVs for various flavor amino acids were umami amino acids > sweet amino acids > bitter amino acids > aromatic amino acids. The total content of amino acids in the summer leaves of *I. vomitoria* was 1458.48 µg/g, with the highest content of Asp at 324.17 µg/g. Its TAV was also the highest at 10.81, indicating that it contributes the most to the presentation of amino acid flavor in the summer leaves of *I. vomitoria*. The amino acids that present a fresh flavor in the summer leaves of *I. vomitoria* mainly include Glu and Asp, so these two free amino acids were the main amino acids that present a unique flavor in the summer leaves of *I. vomitoria*.

In autumn, the order of total TAVs for various flavor amino acids was umami amino acids > bitter amino acids > sweet amino acids > aromatic amino acids. The total content of amino acids in the autumn leaves of *I. vomitoria* was 526.8 µg/g, with Ile having the highest content at 116.34 µg/g. However, its TAV was not the highest, with Glu having the highest TAV at 1.8. Therefore, Glu contributes the most to the presentation of amino acid flavor in the autumn leaves of *I. vomitoria*. The main amino acids that present a bitter taste in the autumn leaves of *I. vomitoria* were Arg, and the main amino acids that present a fresh taste were Glu. Therefore, these two free amino acids were the main amino acids that present a unique flavor in the autumn leaves of *I. vomitoria*.

### 2.4. Analysis of Antioxidant Content and Antioxidant Activity in Leaves of I. vomitoria

According to Table 4, the leaf flavonoid content was highest in spring and significantly greater than in summer and autumn (*p* < 0.05), with the lowest levels observed in autumn. The flavonoid content in spring was 38% and 208% higher than in summer and autumn, respectively. The leaf polyphenol content was highest in spring and significantly greater than in summer and autumn (*p* < 0.05), with the lowest levels observed in autumn. The polyphenol content in spring was 33% and 77.7% higher than in summer and autumn, respectively. The leaf total saponin content was highest in summer and significantly greater than in spring and autumn (*p* < 0.05), with the lowest levels observed in autumn. The polyphenol content in summer was 11% and 31% higher than in spring and autumn, respectively. The leaf caffeine content was highest in spring and significantly greater than in summer and autumn (*p* < 0.05), with the lowest levels observed in autumn. The caffeine content in spring and summer was 219.97% and 206.21% higher than in autumn, respectively. The leaf catechins content was highest in spring and significantly greater than in summer and autumn (*p* < 0.05), with the lowest levels observed in autumn. The catechins content in spring was 5 times and 9 times higher than in summer and autumn, respectively.

DPPH is a synthetic organic free radical commonly used to determine the antioxidant activity of plants. According to Table 5, the DPPH free radical scavenging ability of leaves gradually decreased with seasonal changes. The DPPH free radical scavenging ability of spring leaves was the strongest, at 305.58 µmol TE/g, significantly higher than that of summer and autumn, and the lowest in autumn, at 96.84 µmol TE/g. The DPPH free radical scavenging ability in spring was 1.08 and 3.15 times that of summer and autumn, respectively.

ABTS cations are oxidized by oxidants such as K_2_S_2_O_8_ to produce blue-green ABTS cationic free radicals. The absorbance of the reaction mixture at a specific wavelength (typically 734 nm), which is directly proportional to the ABTS^+^ concentration, is measured to assess antioxidant capacity of the sample. The ABTS^+^ free radical scavenging ability of spring leaves was determined to be 43.04 µmol TE/g, and the lowest in autumn was 36.72 µmol TE/g.

The reducing ability of *I. vomitoria* leaves was determined using the FRAP iron ion reducing ability method. This method determines the reduction ability of leaves based on the absorbance when Fe^3+^ is reduced to Fe^2+^ by antioxidant substances. The higher the absorbance value, the stronger the reducing ability. From Table 5, it can be seen that the FRAP iron ion reducing ability of leaves gradually decreases with seasonal changes in the three seasons. The metal ion reducing capacity in spring leaves was the strongest, at 375.23 µmol TE/g, significantly higher than that in summer and autumn (*p* < 0.05), and the lowest in autumn, at 329.54 µmol TE/g. The metal ion reducing capacity in spring was 9.41% and 13.86% stronger than that in summer and autumn, respectively.

As shown in Table 6, by comparing various correlation factors, it can be seen that in spring, the content of polyphenols, flavonoids, caffeine, catechins, and saponins in the leaves of *I. vomitoria* was significantly more correlated with DPPH free radical scavenging ability, ABTS cation free radical scavenging ability, and FRAP iron ion reducing ability than in summer and autumn. In spring leaves, flavonoids had the highest correlation with ABTS cation free radical scavenging ability, polyphenols had the highest correlation with FRAP iron ion reducing ability, and flavonoids had the highest correlation with DPPH free radical scavenging ability, with correlation coefficients of 0.996, 0.985, and 0.999, respectively. It can be inferred that the strongest ABTS cation free radical scavenging ability and DPPH free radical scavenging ability in spring may be related to the highest flavonoid content in spring.

### 2.5. Analysis of Aroma Components in Leaves of Different Seasons of I. vomitoria

Seven types of aromatic compounds were isolated from the leaves of *I. vomitoria*, mainly including alcohols, ketones, and aldehydes, with average contents of 58.70%, 29.75%, and 27.50%, respectively. The remaining components were esters (6.40%), other types (ether, benzene, alkane) (5.83%), olefins (4.76%), and acids (1.38%). The relative content of various aroma components in leaves varied greatly in different seasons, with the proportion of alcohol, ketone, aldehyde, lipid, other types, alkene, and acid aromas reaching the highest level in spring (Table 7).

A total of 82 aroma components were reported in the leaves of *I. vomitoria* in Table 8. In spring, the highest content of aroma compounds in the leaves of *I. vomitoria* were alcohol compounds, accounting for 66.08%. In addition, ketones and aldehydes account for 48.07% and 38.51%, respectively. Esters, other compounds (ether, benzene, alkane), alkenes, and acids account for 9.91%, 9.00%, 6.58%, and 1.63%, respectively. Among the alcohols, (2E)-3,7-dimethylocta-2,6-dien-1-ol was the predominant compound, which was 14.06%. The contents of 2,6-dimethylcyclohexan-1-ol, 2-ethylhexan-1-ol, pentan-1-ol, hept-6-en-1-ol, and butan-1-ol reached 13.62%, 13.06%, 12.77%, 11.26%, and 11.21%, respectively; the highest content of ketone compounds was 1-hydroxypropan-2-one, with a content of 12.10%. octan-2-one, heptan-2-one, hept-1-en-3-one, and hexane-2,3-dione reach 11.46%, 7.63%, 5.56%, and 4.95%, respectively; the 2-phenylacetaldehyde with the highest content in aldehyde compounds reached 14.29%, while hexanal, (E)-hex-2-enal, (E)-pent-2-enal, (E)-hept-4-enal, and (E)-hex-3-enal reached 7.76%, 4.95%, 2.65%, 1.52%, and 1.45%, respectively.

In summer, the highest content of aromatic compounds in the leaves of *I. vomitoria* was alcohol compounds, accounting for 55.04%, followed by aldehydes and ketones, accounting for 22.74% and 17.63%, respectively. The content of other compounds, esters, alkenes, and acids was 4.91%, 4.89%, 2.99%, and 1.22%, respectively. The highest content of alcohol compounds was 2-ethylhexan-1-ol, accounting for 25.12%. The contents of butan-1-ol, (2E)-3,7-dimethylocta-2,6-dien-1-ol, and pentan-1-ol reached 16.26%, 13.68%, and 12.41%, respectively. The highest content of ketones in summer was octan-2-one, which accounts for 5.32%, while the content of other ketones was relatively low. The aldehyde compounds with higher content were 2-phenylacetaldehyde, which accounts for 11.08%, and hexanal, which accounts for 4.43%.

In autumn, the highest content of aromatic compounds in the leaves of *I. vomitoria* was also alcohol compounds, accounting for 54.98%, followed by ketone compounds and aldehyde compounds, with contents of 23.57% and 21.27%, respectively. The contents of other compounds, alkenes, esters, and acids were 4.90%, 4.72%, 4.40%, and 1.03%, respectively. The highest content in alcohol compounds was (2E)-3,7-dimethylocta-2,6-dien-1-ol, which was 18.53%, followed by 2-ethylhexan-1-ol with a content of 13.35%; the highest content of ketone compounds in autumn leaves was 1-hydroxypropan-2-one, accounting for 10.86%, followed by octan-2-one, accounting for 9.21%; the highest content of aldehydes in autumn leaves was hexanal, with a content of 10.66%.

In summary, the content of alcohols, aldehydes, esters, other compounds, and acids in the leaves of *I. vomitoria* was in the order of spring>summer>autumn, while the content of ketones and alkenes was in the order of spring>autumn>summer. Spring was the season with the highest proportion of most aromatic substances, followed by summer, and finally autumn.

## 3. Discussion

The content of N and Fe in leaves of *Ilex vomitoria* showed an overall decreasing trend with seasonal changes, while the content of Ca showed an increasing trend, and the content of P, Mg, and Zn showed a first decreasing and then increasing trend. The contents of N and P in spring leaves of *I. vomitoria* leaves were significantly higher than those in summer and autumn, and our findings are consistent with a recent study [13]. Research has shown that N absorption is closely related to the active growth of young tea buds [14]. High content of N significantly increases tea yield and the levels of polyphenols and caffeine [15,16]. P has been proven to alter the metabolism of minerals and metabolites in tea plants, thus affecting tea yield and quality [17]. P contributes to improving the aroma and flavor of tea [18]. Both N and P increase can enhance tea yield and growth. N and P are also essential elements in the formation of enzymes like nitrate reductase (NR), adenosine triphosphate (ATP), and nicotinamide adenine dinucleotide phosphate (NADP), thereby boosting the antioxidant activity of tea [19]. The content of Mg and Fe in the leaves of *I. vomitoria* in spring was the highest, and higher than that in the *I. paraguariensis* A.St.-Hil. [20]. Mg is an essential component of chlorophyll, which plays a crucial role in photosynthesis and many other metabolic processes. [21]. The content of Mg was highest in spring, coinciding with the highest synthesis of carbohydrates through photosynthesis, resulting in the highest levels of soluble sugars [22]. Fe is involved in key processes such as DNA synthesis, respiration, photosynthesis, and nitrogen degradation [23]. Studies have shown that increased Ca supply induces stomatal closure and mediates stress responses, aiding in cold injury recovery and adaptation to cold stress [24]. In this study, the highest content of Ca was observed in the autumn, likely reflecting physiological adjustments to cooler temperatures. Research has also shown that Zn is related to the amino acid content in tea leaves [25]. Zn plays a critical role in nucleic acid and protein synthesis and aids in the utilization of N and P [26]. Compared to summer and autumn tea, spring tea leaves contain higher levels of essential mineral nutrients, thus leading to higher concentrations of amino acids, polyphenols, and caffeine—all of which contribute to better tea quality [27]. Spring provides favorable weather conditions with steadily rising temperatures and high humidity, ideal for the growth of new tea buds [28]. Therefore, considering these factors, spring tea leaves of *I. vomitoria* have the richest nutritional content and are recommended for further development and utilization.

Soluble sugars are important carbohydrates in photosynthesis and are also one of the consumables in respiration. Tea leaves predominantly accumulate sugar conjugates, whose biosynthesis is closely linked to N metabolism. N is essential for chlorophyll’s porphyrin structure, and enhances photosynthetic efficiency to drive sugar production via the Calvin cycle, serving as precursors for sugar conjugates [29]. The content of N in the leaves of *I. vomitoria* is highest in spring, which corresponds to the highest levels of soluble sugars synthesized. Soluble sugars are key determinants of the sweet taste quality in tea [30]. In this experiment, the soluble sugar content in the leaves of *I. vomitoria* in spring was significantly higher than the other two seasons.

The vitamin C content in the leaves of *I. vomitoria* varied with the seasons. This study showed that the highest vitamin C content occurs in autumn. Based on these experimental results, we would recommend harvesting leaves in autumn for the preparation of *I*. *vomitoria* tea and its development into health beverages, due to its higher water-soluble vitamin C content. In addition, vitamin C is a natural oxidant with a natural oxidizing effect, which can be developed and utilized into compound medicines and beauty and skincare products [31].

The unique flavor, quality, and health benefits of tea are closely linked to a variety of secondary metabolites. Key secondary metabolites influencing tea quality include polyphenols, total amino acids, and aroma compounds. Among them, polyphenols and amino acids directly affect the taste, while polyphenol oxidation products contribute to the color of tea, and aroma compounds determine its fragrance quality. The synthesis of these metabolites is regulated during different growth stages of tea plants [32]. Seasonal climatic conditions have a significant impact on the quality of tea before harvest. For instance, spring tea is obviously higher in quality compared to summer or autumn tea [33]. The differences in quality between spring and summer teas are primarily due to variations in environmental conditions, which affect the synthesis of secondary metabolites [34,35].

The content of free amino acids is considered an important indicator for ensuring tea quality, as they contribute to the overall taste and color [36]. High amino acid levels increase the freshness of tea infusions [32]. In spring, the total amount of free amino acids in the leaves of *I. vomitoria* is the highest, higher than the summer congou black tea [37], and much higher than the amino acid content of *C. sinensis* tea [38], and is consistent with the findings in green tea [39]. N is essential for the biosynthesis of amino acids, and increased N content may help elevate the total amino acid levels in tea leaves [40]. Accordingly, our study found the highest N and total amino acid contents in the leaves of *I. vomitoria* in spring.

From a flavor perspective, amino acids play a crucial role as they are essential substances that contribute to aroma and flavor. According to the different taste characteristics of amino acids, they are divided into four types: fresh, sweet, bitter, and aromatic. Due to the relatively low abundance and higher detection threshold of bitter amino acids, they generally do not contribute to tea flavor [39]. Studies suggest that Glu and Asp contribute to the umami taste [41]. Sweet amino acids and umami amino acids have a synergistic effect [42], masking bitterness while also increasing aroma and umami taste. In this study, the highest contents in summer leaves of *I. vomitoria* were Glu and Asp, and these two umami amino acids contribute to enhancing their nutritional and flavor quality. Consequently, the summer leaves exhibited the highest amino acid nutritional quality among tested seasons. From the perspective of amino acid nutritional quality, it is recommended to utilize summer leaves for developing and processing high-umami flavored tea, theanine-functional beverages, natural umami enhancers, and amino acid-fortified foods.

The overall trend of the content of flavonoids, polyphenols, caffeine, and catechins in the leaves of *I. vomitoria* shows that the content is highest in spring. In spring, the leaves of *I. vomitoria* have stronger DPPH free radical scavenging ability, ABTS cation free radical scavenging ability, and FRAP iron ion reducing ability than in summer and autumn, which may be related to the fact that spring leaves contain the highest antioxidant active substances such as flavonoids, polyphenols, catechins and caffeine [43,44].

Flavonoids are known for their therapeutic effects on various conditions such as cancer, atherosclerosis, and Alzheimer’s disease [45,46], which plays an important role in plant defense against oxidative stress. They serve as UV filters, protecting plants from various biotic and abiotic stresses, acting as signaling molecules, detoxifying agents, and antimicrobial compounds, and are responsible for the color and fragrance of fruits and flowers [47]. The highest flavonoid content in spring leaves of *I. vomitoria* indicates strong antioxidant properties and positions these leaves as a promising plant for medical use. Polyphenols, another crucial component in tea, are regarded as important compounds contributing to the health benefits of tea [48]. They are also considered the most significant antioxidants [49]. The antioxidant activity is well correlated with the total polyphenol content in tea, and teas with higher polyphenol levels exhibit stronger antioxidant activities. As an essential mineral nutrient for plants, P indirectly participates in the synthesis and activity regulation of phenylalanine ammonia-lyase (PAL) by modulating the transcription or translation processes of the PAL gene, thereby influencing the biosynthesis of polyphenolic compounds in plants such as tea trees. [17]. Therefore, the highest content of P in the leaves of *I. vomitoria* in spring may lead to the highest polyphenol levels and the strongest antioxidant activity. Saponins have been increasingly recognized for their anticancer, antiviral, antibacterial, anti-inflammatory, anti-Alzheimer’s, antioxidant, and immunomodulatory activities, as well as their ability to inhibit α-glucosidase [50]. This suggests that teas with higher saponin content may provide more health benefits [51]. In this study, the summer leaves of *I. vomitoria* were found to have the highest saponin content. Therefore, developing and processing these leaves into functional foods or dietary supplements rich in saponins represents a promising approach to utilize their bioactive composition for promoting human health. Caffeine exhibits blood pressure-lowering and anxiolytic effects [32]. Studies have also shown that increased nitrogen metabolism in spring is beneficial for caffeine synthesis, resulting in significantly higher caffeine content in spring tea compared to summer tea [32], consistent with our findings. Catechins, which contribute to the bitterness and astringency of tea, are essential for tea’s biological activity and health benefits [52]. They are involved in a variety of pharmacological actions, particularly their powerful antioxidant properties [53,54]. In this study, the spring leaves of *I. vomitoria* had the highest levels of antioxidant compounds, suggesting that spring tea leaves are the most suitable for development and utilization. Based on these findings, potential applications include the formulation of antioxidant functional beverages, tea polyphenol-enriched functional foods, antioxidant probiotic formulations, and antioxidant skincare products, leveraging the antioxidant activity of these compounds for health and cosmetic purposes.

GC-MS technology was used in this study to analyze and identify the aroma components in the leaves of *I. vomitoria* in different seasons. It was found that the aroma components in the leaves *I. vomitoria* in spring, summer, and autumn were mainly composed of alcohols, ketones, and aldehydes. This is consistent with the results of the aroma components of *Viscum articulatum* Burm.f. [55]. The aroma components such as alcohols, ketones, and aldehydes in the leaves are highest in spring, which is inconsistent with the results of previous studies on black tea [56,57]. This may be attributed to the significant influence of cultivars [58] and production seasons [59] on the volatile compounds in tea leaves. Climatic conditions during different production seasons, particularly significant differences in temperature and sunlight [60,61], lead to substantial variations in the aroma components of tea leaves [60]. Alcohols dominate in spring leaves, especially (2E)-3,7-dimethylocta-2,6-dien-1-ol (14.06%), 2,6-dimethylcyclohexan-1-ol (13.62%), 2-ethylhexan-1-ol (13.06%), and hept-6-en-1-ol (11.26%). The formation of alcohols is primarily related to the hydrolysis of glycosidic precursors and the biosynthesis of volatile terpenoids, which significantly influence the fragrance quality of tea [62,63]. Among these, (2E)-3,7-dimethylocta-2,6-dien-1-ol is the most common alcohol in tea and is considered a key aromatic compound responsible for the floral, fruity, and sweet notes in tea’s aroma [64,65]. (2E)-3,7-dimethylocta-2,6-dien-1-ol also has antiviral, antibacterial, and anti-inflammatory effects and is very helpful in improving blood circulation and regulating glucose and lipid metabolism in the human body [66,67]. At the same time, it was found that the aroma components of 2,6-dimethylcyclohexan-1-ol with rose and citrus aromas were higher in spring leaves. Therefore, overall, the spring leaves of *I. vomitoria* have the most abundant aroma components.

## 4. Materials and Methods

### 4.1. Materials

The plant material used in this study was *Ilex vomitoria*, which was cultivated by the experimenters and sourced from the *Ilex* plants nursery of Zhejiang A&F University. It was identified by plant classification experts Yan Daoliang and Lou Luhuan. Six-year-old *I*. *vomitoria* plants were selected, which were planted in organic yellow soil with a pH 6~6.5. Tender leaves were picked which contained one bud one leaf in spring (April), mature leaves in summer (July), and old leaves in autumn (October), placed in a low-temperature box, and quickly brought back to the laboratory. After freeze-drying and crushing with liquid nitrogen, they were used to detect the content of mineral nutrients, soluble sugar, amino acids, and antioxidant substances in the leaves, and we analyzed indicators such as antioxidant activity. At the same time, the vitamin C content and aroma components of fresh leaves during the same period were measured.

### 4.2. Methods

#### 4.2.1. Determination of Nutrient Elements in Leaves

The nitrogen (N) content in the leaves was determined using the Kjeldahl method. First, the leaf powder (0.2 ± 0.001 g) was placed into a 100 mL digestion tube. CuSO_4_·5H_2_O was used as a Kjeldahl catalyst, followed by 8 mL of 98% concentrated sulfuric acid. The leaf powder was digested at 280 °C for 60 min using a graphite digestion instrument. After cooling, 2 mL of hydrogen peroxide was added, and the tube was returned to the digestion instrument, where it was heated at 300 °C for 60 min until the solution turned a brown-black color. The tube was removed, cooled, and the nitrogen content was measured using an automatic Kjeldahl nitrogen analyzer (Hanon K9860, Hanon Advanced Technology Group Co., Ltd, Jinan, China).

For phosphorus(P) content determination, the molybdenum–antimony colorimetric method was adopted. The leaf powder (0.5 g) was placed into a 50 mL digestion tube. To this, 2 mL of 68% concentrated nitric acid, 0.5 mL of hydrogen peroxide, and 0.5 mL of deionized water were added. The leaf powder underwent pre-reaction for 1 h, and the leaf powder and solution in the digestion tube were mixed and then left overnight. The leaf powder was heated in a digestion furnace, rising to 250 °C within 25 min, followed by a 15 min incubation at 250 °C. Afterward, the sample was cooled rapidly for 20 min. The excess acid solution was removed using a graphite heater at 160 °C, and the sample was cooled to room temperature. After rinsing with deionized water and diluting to 25 mL, the solution was clarified and the phosphorus content was determined by measuring the absorbance at 700 nm using a UV spectrophotometer (UV754N, Shanghai INESA Analytical Instrument Co., Ltd., Shanghai, China).

For potassium(K), magnesium(Mg), calcium(Ca), iron(Fe), and zinc(Zn) content determination, 0.5 g of the leaf powder was placed in a PTFE digestion tube. To this, 90 mL 0.80 mol/L nitric acid and 40 mL 1.05 mol/L perchloric acid were added. The mixture was heated until red fumes dissipated. After cooling, 20 mL of deionized water was added, and the solution was filtered for atomic absorption spectroscopy, which following the method described by Albakaa et al. [68].

#### 4.2.2. Determination of Soluble Sugar and Vitamin C in Leaves

For soluble sugar determination, 0.5 g of dry leaf powder was mixed with 1 mL of 80% ethanol and ethanol to form a paste. The paste was transferred to a 10 mL centrifuge tube and subjected to a water bath at 100 °C for 20 min. After cooling, the leaf powder was centrifuged at 8000 rpm for 30 min at room temperature, and the supernatant absorbance was measured at 620 nm using a microplate reader (Thermo Fisher Scientific, Waltham, MA, USA).

For vitamin C determination, 0.1 g of the leaf powder was placed into a centrifuge tube and 5 mL of 2% oxalic acid-EDTA buffer solution (pH 4.0) was added. The leaf powder was boiled in a water bath for 15 min, then cooled to room temperature. After centrifuging at 8000 rpm for 25 min, 1 mL of the supernatant was transferred to a clean test tube. To this, 2 mL of oxalic acid–EDTA buffer, 1 mL of 2% phosphoric acid–acetic acid solution, 2 mL of 5% sulfuric acid solution, and 1 mL of 5% ammonium molybdate solution were added. The mixture was shaken, incubated in a water bath at 37 °C for 25 min, and then 1.5 mL of deionized water was added. The absorbance was recorded at 760 nm, and the vitamin C content was calculated using the corresponding formula [69].

#### 4.2.3. Determination of Amino Acids in Leaves

One gram of the leaf powder was weighed, and 25 mL of 0.1% phenol solution and 5 mol/L hydrochloric acid were added for grinding. After evaporating the solvent, 2 mL of 0.1 M hydrochloric acid solution was added, and the mixture was filtered. A 100 µL aliquot of the filtrate and a 100 µL aliquot of amino acid standard solution were placed into a 2 mL EP tube. To the tube, 20 µL of 0.05 mol/L N-Leucine Internal Standard Solution, 100 µL of 1.0 mol/L triethylamine acetonitrile solution (ensuring pH 7), and 100 µL of 0.2 mol/L phenyl isothiocyanate acetonitrile solution were added. The mixture was shaken and allowed to stand for 1 h at 25 °C. Afterward, 1 mL of 95% n-hexane was added, the solution mixed, and allowed to stand for 10 min. The aqueous lower phase (containing phenylthiocarbamyl amino acid derivatives) was then diluted five times, filtered through a 0.22-µm membrane, and analyzed.

The amino acids were detected using HPLC analyzer (Agilent 1100, Agilent Technologies, Inc., Santa Clara, USA) equipped with Compass C18 column (250 mm × 4.6 mm, 5 µm, Suzhou Saifen Technology Co., Ltd, Suzhou, China) [70]. The injection volume was 10 µL and column temperature was 40 °C. Mobile phase A was a mixture of 6.6 g anhydrous sodium acetate, 950 mL distilled water, 70 mL glacial acetic acid, and acetonitrile, and mobile phase B was 80% acetonitrile aqueous solution. The linear gradient of the solvent was 0–2 min, 100% A; 2–15 min, 100–90% A; 15–25 min, 90–70% A; 25–33 min, 70–55% A; 33–33.1 min, 50–0% A; 33.1–38 min, 0% A; 38–38.1 min, 0–100% A; and 38.1–45 min, 100% A. The flow rate was 1 mL/min. The ultraviolet absorption wavelength was 254 nm. The standard curve was constructed according to the peak area and concentration, and the concentration was calculated.

The flavor intensity of amino acids was evaluated based on the taste active value (TAV), calculated as the ratio of a specific amino acid content to its taste threshold. If TAV ≥ 1, the amino acid contributed to the overall flavor; if TAV <  1, it was considered non-contributory [71].

#### 4.2.4. Determination of Flavonoids and Polyphenols in Leaves

Flavonoids and polyphenols were extracted from 0.4 g of the leaf powder using 20 mL of ethanol solution by ultrasonic extraction. The measurement method followed the procedure described by TranThi et al. [72].

#### 4.2.5. Determination of Saponins in Leaves

First, 0.5 g of dry leaf powder was mixed with water and allowed to stand for 30 min. The mixture was filtered through a multilayer gauze, centrifuged at 1000 rpm for 15 min, and 0.2 mL of 5% vanillin–acetic acid solution and 0.8 mL of 70% perchloric acid were added to the supernatant. The solution was extracted at 50 °C for 2 h, and the resulting supernatant was reserved for analysis. The content of saponins was determined by measuring the absorbance at 550 nm, using oleanolic acid standard solutions ranging from 8 to 40 μg/mL.

#### 4.2.6. Determination of Caffeine and Catechins in Leaves

Approximately 0.1 g of the leaf powder was weighed and placed into a 2 mL centrifuge tube. Then, 1.0 mL of 70% methanol which was preheated to 70 °C was added, and the mixture was subjected to a 70 °C water bath for 30 min. Afterward, the leaf powder was centrifuged, and the supernatant was collected. An additional 1.0 mL of 70% methanol was used for extraction, and the supernatants were combined and made up to 2 mL with 70% methanol. The solution was then filtered using a syringe filter (0.22 µm) and was ready for analysis. The catechins and caffeine were detected using a HPLC analyzer (Waters 2695, Suzhou Leiden Scientific Instrument Co., Ltd., Suzhou, China) equipped with a Compass C18 column (250 mm × 4.6 mm, 5 µm). The injection volume was 10 µL and column temperature was 35 °C. Mobile phase A consisted of DMF–methanol–glacial acetic acid in a ratio of 40:2:1.5, and mobile phase B was water. The linear gradient of the solvent was 0–10 min, 9–14% A; 10–15 min, 14–23% A; 15–27 min, 23–36% A; 27–35 min, 36–50% A; 35–36 min, 50–9% A; and 36–45 min, 9% A. The flow rate was 1 mL/min. The absorbance of caffeine and catechins was measured at 278 nm, using caffeine standard solutions in the range of 1–400 μg/mL and gallic acid standard solutions in the range of 1–200 μg/mL, respectively.

#### 4.2.7. Determination of Antioxidant Capacity in Leaves

The antioxidant capacity of the leaves was measured by DPPH radical scavenging, ABTS cation radical scavenging, and the ferric reducing antioxidant power (FRAP) methods, as described by TranThi et al. [72]. 0.1 g of the leaf powder was mixed with 1 mL extraction solution, ground in an ice bath, and then centrifuged at 12,000 rpm for 10 min at 4 °C. The supernatant was used for further analysis.

#### 4.2.8. Determination of Aroma Components in Leaves

Fresh leaves were ground into a fine powder and 3 g of the leaf powder was accurately weighed and transferred into a 20 mL headspace vial. The leaf powder was then incubated in a 60 °C water bath for 5 min, followed by the addition of 65 µL of 2-octanol. After sealing, static headspace extraction was performed in the water bath for 50 min, then the extract was collected for analysis.

The aroma components in the leaves were determined by gas chromatography–mass spectrometry (Varian 450-GC/240-MS, Varian, Inc., Palo Alto, USA) with manual injection. The gas chromatograph (GC) conditions were as follows: The leaf powder inlet and detector temperatures were set to 250 °C. High-purity helium was used as the carrier gas with a flow rate of 1.5 mL/min. The initial column temperature was set to 50.5 °C, which was then gradually increased to 210 °C. This temperature was maintained for 3 min before being raised to 230 °C. The mass spectrometry (MS) conditions were as follows: The ion source temperature was maintained at 230 °C, with an electron energy of 70 eV and an emission current of 34.6 µA. The quadrupole temperature was set to 150 °C, while the switch interface temperature was 280 °C. The electric multiplier voltage was adjusted to 350 V, and the mass scan range was between 35 and 400 amu.

#### 4.2.9. Statistical Analysis

All experiments were expressed as means ± standard deviation of triplicate measurements and significance analysis was performed by SPSS 26.0 software. Analysis of variance (ANOVA) was carried out by Dunnett’s test, where *p* < 0.05 was assumed to be statistically significant.

## 5. Conclusions

This study determined and analyzed the nutrient elements, antioxidant substances, antioxidant capacity, and aroma components of the leaves of *Ilex vomitoria*. The results showed that except for the highest content of vitamin C in autumn leaves and the highest content of saponins in summer, all other substances had the highest content in spring. The contents of mineral elements, soluble sugars, amino acids, flavonoids, polyphenols, caffeine, and catechins in the leaves of *I. vomitoria*, as well as the DPPH free radical scavenging ability, ABTS cation free radical scavenging ability, and FRAP iron ion reducing ability, were all the highest in spring. A comprehensive evaluation indicated that the leaves in spring are more suitable for picking, development, and utilization. The findings of this study provide critical insights for guiding the development and utilization of *I. vomitoria* leaves, which can be further processed into health drinks, antioxidant skincare products, and medicinal health foods for human consumption.

## Figures and Tables

**Table 1:** Content of mineral nutrient elements and nutritional components in leaves of *Ilex vomitoria* in different seasons ($\bar{x}$ ± *s*, *n* = 3).

Season	Macroelement (g/kg)					Microelement (mg/kg)		Soluble Sugar (mg/g)	Vitamin C (mg/g)
	N	P	K	Mg	Ca	Fe	Zn		
Spring	17.00 ±0.15 a	1.89 ±0.05 a	14.09 ±0.14 a	2.94 ±0.02 a	3.74 ±0.04 b	81.09 ±2.76 a	232.03 ±2.05 a	447.79 ±4.45 a	17.11 ±0.43 c
Summer	11.90 ±0.45 b	0.72 ±0.03 c	13.16 ±0.26 a	1.75 ±0.19 b	4.07 ±0.55 b	66.82 ±2.45 b	182.00 ±24.35 b	269.81 ±0.17 c	22.30 ±0.33 b
Autumn	11.86 ±0.17 b	1.15 ±0.02 b	12.51 ±0.17 a	2.69 ±0.02 a	5.47 ±0.02 a	71.73 ±0.07 b	276.17 ±0.39 a	324.66 ±10.30 b	29.10 ±0.54 a

Note: Different lowercase letters indicate the differences in element content among seasons (*p* < 0.05).

**Table 2:** Amino acid of *Ilex vomitoria* leaves in different seasons.

Amino Acid	Content(µg/g)		
	Spring	Summer	Autumn
Asp	72.87 ± 7.92 b	324.17 ± 6.66 a	21.88 ± 3.63 c
Glu	157.45 ± 5.72 b	323.71 ± 4.59 a	89.85 ± 1.95 b
Ser	36.62 ± 9.51 ab	66.56 ± 1.41 a	11.22 ± 0.91 b
Gly	39.68 ± 2.06 a	12.15 ± 0.83 b	9.378 ± 0.82 b
His	28.70 ± 3.10 b	121.97 ± 1.67 a	9.28 ± 0.66 b
Arg	402.81 ± 6.85 a	16.31 ± 0.28 c	106.76 ± 0.33 b
Thr	37.33 ± 0.82 a	33.02 ± 1.86 b	9.39 ± 0.79 c
Ala	130.62 ± 1.37 a	88.44 ± 3.47 b	36.50 ± 0.79 c
Pro	76.40 ± 3.93 a	23.03 ± 1.01 b	11.71 ± 0.50 c
Tyr	73.06 ± 2.49 a	42.00 ± 2.03 b	17.56 ± 0.76 c
Val	78.62 ± 2.81 a	33.23 ± 1.98 b	14.71 ± 0.37 c
Met	21.62 ± 1.98 a	20.15 ± 1.06 a	9.48 ± 0.05 b
Cys	20.36 ± 1.17 a	11.96 ± 1.12 b	15.09 ± 0.31 a
Ile	169.92 ± 8.81 a	110.05 ± 3.13 b	116.34 ± 4.38 b
Leu	174.84 ± 8.24 a	99.53 ± 3.05 b	41.45 ± 4.31 c
Phe	89.91 ± 3.64 a	61.17 ± 2.59 b	21.81 ± 0.93 c
Lys	36.35 ± 0.50 a	37.66 ± 2.80 a	12.01 ± 0.40 b
Essential amino acid	571.62	394.81	225.19
Total amino acids	1645.14	1425.10	554.81

Note: Different lowercase letters in the same line indicate significant differences. (*p* < 0.05). Asp: Aspartic acid; Glu: Glutamic acid; Ser: Serine; Gly: Glycine; His: Histidine; Arg: Argnine; Thr: Threonine; Ala: Alanine; Pro: Proline; Tyr: Tyrosine; Val: Valine; Met: Methionine; Cys: Cysteine; Ile: Isoleucine; Leu: Leucine; Phe: Phenylalanine; Lys: Lysine.

**Table 3:** Flavor amino acid of *Ilex vomitoria* leaves in spring, summer, and autumn.

Class	Type of Amino Acids	Taste Threshold (µg/g)	Spring		Summer		Autumn	
			Content (µg/g)	TAV	Content (µg/g)	TAV	Content (µg/g)	TAV
Sweet amino acids	Ala	600	130.62	0.3	88.44	0.15	36.50	0.06
	Pro	3000	76.40	0.03	23.03	0.01	11.71	0.01
	His	200	28.70	1.43	121.97	0.61	9.28	0.05
	Thr	2600	37.33	0.01	33.02	0.01	9.39	0.01
	Ser	1500	36.62	0.02	66.56	0.04	11.22	0.01
	Gly	1100	39.68	0.04	12.15	0.01	9.38	0.01
Bitter amino acids	Val	1500	78.62	0.05	33.23	0.02	14.71	0.01
	Leu	3800	174.84	0.04	99.53	0.03	41.45	0.01
	Ile	900	169.92	0.18	110.05	0.12	116.34	0.13
	Met	300	21.62	0.07	20.15	0.07	9.48	0.31
	Thr	2600	37.33	0.01	33.02	0.01	9.39	0.01
	Arg	100	402.81	4.03	16.31	0.16	106.76	1.06
Umami amino acids	Lys	500	36.35	0.07	37.66	0.07	12.01	0.02
	Asp	30	72.87	2.43	324.17	10.81	21.88	0.73
	Glu	50	157.45	3.15	323.71	6.47	89.85	1.8
Aromatic amino acids	Phe	1500	89.91	0.06	61.17	0.01	21.81	0.01
	Tyr	2600	73.06	0.03	42.00	0.02	17.56	0.01
	Cys	20	20.36	1.01	11.96	0.05	15.09	0.01
Total	-	-	1601.13	-	1458.48	-	526.8	-

Note: Asp: Aspartic acid; Glu: Glutamic acid; Ser: Serine; Gly: Glycine; His: Histidine; Arg: Argnine; Thr: Threonine; Ala: Alanine; Pro: Proline; Tyr: Tyrosine; Val: Valine; Met: Methionine; Cys: Cysteine; Ile: Isoleucine; Leu: Leucine; Phe: Phenylalanine; Lys: Lysine.

**Table 4:** Physiologically active substance contents of *Ilex vomitoria* leaves in different seasons.

Season	Flavonoid (mg/g)	Polyphenol (mg/g)	Saponins (mg/g)	Caffeine (µg/g)	Catechins (µg/g)
Spring	64.86 ±1.10 a	35.45 ±0.34 a	130.45 ±6.95 ab	2982.30 ±41.07 a	1083.84 ±37.53 a
Summer	46.48 ±0.28 b	26.64 ±1.68 b	144.56 ±13.06 a	2853.98 ±33.07 a	215.31 ±18.32 b
Autumn	20.40 ±0.47 c	19.94 ±0.49 c	110.10 ±1.06 b	932.03 ±93.05 b	115.17 ±1.38 c

Note: Different lowercase letters in the same column indicate significant differences. (*p* < 0.05).

**Table 5:** Antioxidant evaluation method of *Ilex vomitoria* leaves in different seasons.

Season	DPPH Free Radical Scavenging Ability (µmol TE/g)	ABTS+ Free Radical Scavenging Ability (µmol TE/g)	FRAP Iron Ion Reducing Ability (µmol TE/g)
Spring	305.58 ± 9.73 a	43.04 ± 1.93 a	375.23 ± 2.82 a
Summer	146.85 ± 3.24 b	42.23 ± 0.51 a	342.95 ± 4.90 c
Autumn	96.84 ± 10.50 c	36.72 ± 0.39 b	329.54 ± 3.50 b

Note: Different lowercase letters indicate the differences in element content among seasons (*p* < 0.05).

**Table 6:** Correlation analysis between components of *Ilex vomitoria* leaves in different seasons.

Correlation Factors	ABTS			FRAP			DPPH		
	Spring	Summer	Autumn	Spring	Summer	Autumn	Spring	Summer	Autumn
Caffeine	0.956 **	0.946 **	0.947 **	0.955 **	0.954 **	0.832 **	0.953 **	0.976 **	0.859 **
Catechins	0.976 **	0.937 **	0.954 **	0.975 **	0.956 **	0.954 **	0.975 **	0.955 **	0.943 **
Polyphenol	0.989 **	0.969 **	0.979 **	0.985 **	0.988 **	0.973 **	0.988 **	0.976 **	0.974 **
Flavonoid	0.996 **	0.975 **	0.969 **	0.984 **	0.983 **	0.974 **	0.999 **	0.998 **	0.995 **
Saponins	0.865 **	0.863 **	0.820 **	0.893 **	0.853 **	0.840 **	0.854 **	0.831 **	0.820 **
DPPH	0.885 **	0.856 **	0.856 **	0.775 *	0.735 *	0.765 *			
FRAP	0.893 **	0.913 **	0.934 **						
ABTS									

Note: ABTS represent ABTS cationic free radical scavenging ability, FRAP represent FRAP iron reducing ability, DPPH represent free radical scavenging ability. Asterisks (*, **) denote statistical significance of mean differences among seasons, corresponding to *p* < 0.05 and *p* < 0.01 levels, respectively.

**Table 7:** Composition and relative content of aroma components (%) of *Ilex vomitoria* leaves in different seasons.

Season	Alcohols	Ketones	Aldehydes	Esters	Other Types	Olefins	Acids
Spring	66.08	48.07	38.51	9.91	9.00	6.58	1.63
Summer	55.04	17.63	22.74	4.89	4.91	2.99	1.22
Autumn	54.98	23.57	21.27	4.40	4.90	4.72	1.03
Average contents	58.70	29.75	27.50	6.40	5.83	4.76	1.38

**Table 8:** Analysis of the differences in aroma components of *Ilex vomitoria* leaves in different seasons.

Serial Number	CAS	The Name of the Compound	Molecular Formula	Relative Content(%)			R.T. (Minutes)
				Spring	Summer	Autumn	
1	87-44-5	4,11,11-trimethyl-8-methylidenebicyclo [7.2.0]undec-4-ene	C_15_H_24_	0.19	0.16	0.16	20.894
2	78-85-3	2-methylprop-2-enal	C_4_H_6_O	2.43	0.68	1.60	2.643
3	55683-21-1	3,4,5-trimethylcyclopent-2-en-1-one	C_8_H_12_O	0.39	0.27	0.34	3.387
4	80-56-8	2,6,6-trimethylbicyclo [3.1.1]hept-2-ene	C_10_H_16_	2.14	0.95	1.03	5.101
5	106-24-1	(2E)-3,7-dimethylocta-2,6-dien-1-ol	C_10_H_18_O	14.06	13.68	18.53	26.622
6	600-14-6	pentane-2,3-dione	C_5_H_8_O_2_	1.24	1.25	0.90	6.155
7	122-78-1	2-phenylacetaldehyde	C_8_H_8_O	14.29	11.08	10.66	21.807
8	104-93-8	1-methoxy-4-methylbenzene	C_8_H_10_O	0.86	0.09	0.49	11.58
9	71-41-0	pentan-1-ol	C_5_H_12_O	12.77	12.41	8.26	11.951
10	41519-23-7	[(Z)-hex-3-enyl] 2-methylpropanoate	C_10_H_18_O_2_	0.36	0.24	0.28	28.649
11	142-92-7	hexyl acetate	C_8_H_16_O_2_	0.51	0.49	0.50	12.407
12	586-62-9	1-methyl-4-propan-2-ylidenecyclohexene	C_10_H_16_	0.26	0.11	0.08	12.514
13	111-13-7	octan-2-one	C_8_H_16_O	11.46	5.32	9.21	12.725
14	106-72-9	2,6-dimethylhept-5-enal	C_9_H_16_O	1.78	0.38	1.36	14.612
15	4132-48-3	1-methoxy-4-propan-2-ylbenzene	C_10_H_14_O	0.59	0.11	0.44	14.919
16	111-11-5	methyl octanoate	C_9_H_18_O_2_	0.32	0.08	0.19	15.695
17	31081-18-2	3-methyl-5-propylnonane	C_13_H_28_	0.54	0.20	0.19	16.016
18	104-76-7	2-ethylhexan-1-ol	C_8_H_18_O	13.06	25.12	13.35	18.43
19	4117-10-6	hept-6-en-1-ol	C_7_H_14_O	11.26	1.15	4.59	18.922
20	932-66-1	1-(cyclohexen-1-yl)ethanone	C_8_H_12_O	0.25	0.09	0.10	7.913
21	629-50-5	tridecane	C_13_H_28_	2.74	0.96	1.17	13.205
22	5337-72-4	2,6-dimethylcyclohexan-1-ol	C_8_H_16_O	13.62	6.41	7.43	20.98
23	4412-91-3	furan-3-ylmethanol	C_5_H_6_O_2_	1.69	0.39	1.41	22.397
24	106-68-3	octan-3-one	C_8_H_16_O	1.02	0.77	0.50	11.841
25	116-09-6	1-hydroxypropan-2-one	C_3_H_6_O_2_	12.10	3.06	10.86	13.054
26	502-47-6	3,7-dimethyloct-6-enoic acid	C_10_H_18_O_2_	0.89	0.49	0.40	25.29
27	1576-95-0	(Z)-pent-2-en-1-ol	C_5_H_10_O	0.59	0.00	0.49	13.89
28	543-49-7	heptan-2-ol	C_7_H_16_O	0.50	0.30	0.43	13.955
29	4363-93-3	quinoline-4-carbaldehyde	C_10_H_7_NO	0.23	0.12	0.21	37.265
30	13894-63-8	methyl (E)-hex-2-enoate	C_7_H_12_O_2_	2.12	1.72	2.31	12.843
31	591-93-5	penta-1,4-diene	C_5_H_8_	1.25	0.75	1.3	1.631
32	74-93-1	methanethiol	CH_4_S	0.39	0.62	0.40	1.64
33	75-07-0	acetaldehyde	C_2_H_4_O	-	0.12	0.14	1.683
34	110-00-9	furan	C_4_H_4_O	0.02	0.3	0.08	2.03
35	142-82-5	heptane	C_7_H_16_	0.03	0.21	0.63	1.69
36	78-93-3	butan-2-one	C_4_H_8_O	-	-	0.16	2.86
37	111-84-2	nonane	C_9_H_20_	-	-	0.08	2.877
38	75-18-3	methylsulfanylmethane	C_2_H_6_S	0.85	0.31	1.85	1.834
39	123-38-6	propanal	C_3_H_6_O	-	-	1.16	1.999
40	111-65-9	octane	C_8_H_18_	-	0.33	0.59	2.056
41	78-84-2	2-methylpropanal	C_4_H_8_O	-	-	-	2.12
42	107-02-8	prop-2-enal	C_3_H_4_O	0.49	0.31	0.92	2.338
43	123-72-8	butanal	C_4_H_8_O	-	-	0.14	2.603
44	106-61-6	2,3-dihydroxypropyl acetate	C_5_H_10_O_4_	-	1.92	0.02	9.08
45	67-56-1	methanol	CH_4_O	-	-	0.01	2.847
46	96-17-3	2-methylbutanal	C_5_H_10_O	-	0.47	0.15	3.023
47	590-86-3	3-methylbutanal	C_5_H_10_O	-	0.02	0.17	3.081
48	109-86-4	2-methoxyethanol	C_3_H_8_O_2_	0.95	0.01	0.56	3.319
49	64-17-5	ethanol	C_2_H_6_O	0.77	-	-	3.402
50	78-94-4	but-3-en-2-one	C_4_H_6_O	0.14	0.26	-	3.504
51	110-62-3	pentanal	C_5_H_10_O	0.84	0.60	-	4.139
52	124-18-5	decane	C_10_H_22_	0.06	-	-	4.69
53	1629-58-9	pent-1-en-3-one	C_5_H_8_O	0.34	-	0.08	5.077
54	78-92-2	butan-2-ol	C_4_H_10_O	0.28	0.09	-	5.393
55	71-23-8	propan-1-ol	C_3_H_8_O	0.19	-	0.04	5.717
56	872-05-9	dec-1-ene	C_10_H_20_	0.02	0.11	0.17	5.766
57	7452-79-1	ethyl 2-methylbutanoate	C_7_H_14_O_2_	0.14	-	-	6.013
58	66-25-1	hexanal	C_6_H_12_O	7.76	4.43	0.52	6.711
59	497-03-0	(E)-2-methylbut-2-enal	C_5_H_8_O	0.12	0.04	-	6.982
60	1120-21-4	undecane	C_11_H_24_	-	-	0.03	7.104
61	5878-19-3	1-methoxypropan-2-one	C_4_H_8_O_2_	-	0.76	0.43	7.317
62	78-83-1	2-methylpropan-1-ol	C_4_H_10_O	1.37	0.45	-	7.493
63	1576-87-0	(E)-pent-2-enal	C_5_H_8_O	2.65	1.34	0.76	8.041
64	3848-24-6	hexane-2,3-dione	C_6_H_10_O_2_	4.95	0.35	-	8.129
65	4440-65-7	(E)-hex-3-enal	C_6_H_10_O	1.45	0.89	-	8.324
66	6032-29-7	pentan-2-ol	C_5_H_12_O	0.48	0.43	0.06	8.317
67	71-36-3	butan-1-ol	C_4_H_10_O	11.21	16.26	-	8.947
68	616-25-1	pent-1-en-3-ol	C_5_H_10_O	0.78	0.67	-	9.336
69	108-11-2	4-methylpentan-2-ol	C_6_H_14_O	-	1.28	-	9.607
70	110-43-0	heptan-2-one	C_7_H_14_O	7.63	2.49	-	9.67
71	112-40-3	dodecane	C_12_H_26_	-	-	0.42	10.124
72	6728-26-3	(E)-hex-2-enal	C_6_H_10_O	4.95	0.15	0.02	10.75
73	111-90-0	2-(2-ethoxyethoxy)ethanol	C_6_H_14_O_3_	0.78	0.5	0.01	21.516
74	929-22-6	(E)-hept-4-enal	C_7_H_12_O	1.52	2.11	3.46	11.246
75	928-68-7	6-methylheptan-2-one	C_8_H_16_O	2.31	3.08	-	11.339
76	6728-31-0	(Z)-hept-4-enal	C_7_H_12_O	0.25	0.05	0.13	11.415
77	1120-06-5	decan-2-ol	C_10_H_22_O	4.72	2.21	1.39	11.658
78	763-32-6	3-methylbut-3-en-1-ol	C_5_H_10_O	9.65	3.27	0.03	11.833
79	513-86-0	3-hydroxybutan-2-one	C_4_H_8_O_2_	2.28	0.36	1.34	12.683
80	13894-63-8	methyl (E)-hex-2-enoate	C_7_H_12_O_2_	0.03	1.25	-	12.843
81	2918-13-0	hept-1-en-3-one	C_7_H_12_O	5.56	0.35	0.02	13.181
82	4798-45-2	4-methylpent-1-en-3-ol	C_6_H_12_O	0.03	0.21	0.45	13.655

Note: Other categories include ether, benzene, and alkane compounds; - represents undetected.

## Data Availability

This study did not use publicly available data; all data were generated by the experimenter through experimental design and data processing analysis. The voucher specimen of the plant material *Ilex vomitoria* used in this study is stored at the Herbarium of Xishuangbanna Tropical Botanical Garden, Chinese Academy of Sciences (HITBC), with voucher number 103072. The specimen was identified by Zhu Qiugui and Liu Qingwen.
